# Antibiofilm Activity of an Exopolysaccharide from Marine Bacterium *Vibrio* sp. QY101

**DOI:** 10.1371/journal.pone.0018514

**Published:** 2011-04-07

**Authors:** Peng Jiang, Jingbao Li, Feng Han, Gaofei Duan, Xinzhi Lu, Yuchao Gu, Wengong Yu

**Affiliations:** 1 Key Laboratory of Marine Drugs, Chinese Ministry of Education, Ocean University of China, Qingdao, People's Republic of China; 2 Shandong Provincial Key Laboratory of Glycoscience and Glycotechnology, Ocean University of China, Qingdao, People's Republic of China; 3 Laboratory of Glycobiology, School of Medicine and Pharmacy, Ocean University of China, Qingdao, People's Republic of China; 4 Key Laboratory For Space Bioscience and Biotechnology, Faculty of Life Sciences, Northwestern Polytechnical University, Xi'an, People's Republic of China; University of Osnabrueck, Germany

## Abstract

Bacterial exopolysaccharides have always been suggested to play crucial roles in the bacterial initial adhesion and the development of complex architecture in the later stages of bacterial biofilm formation. However, *Escherichia coli* group II capsular polysaccharide was characterized to exert broad-spectrum biofilm inhibition activity. In this study, we firstly reported that a bacterial exopolysaccharide (A101) not only inhibits biofilm formation of many bacteria but also disrupts established biofilm of some strains. A101 with an average molecular weight of up to 546 KDa, was isolated and purified from the culture supernatant of the marine bacterium *Vibrio* sp. QY101 by ethanol precipitation, iron-exchange chromatography and gel filtration chromatography. High performance liquid chromatography traces of the hydrolyzed polysaccharides showed that A101 is primarily consisted of galacturonic acid, glucuronic acid, rhamnose and glucosamine. A101 was demonstrated to inhibit biofilm formation by a wide range of Gram-negative and Gram-positive bacteria without antibacterial activity. Furthermore, A101 displayed a significant disruption on the established biofilm produced by *Pseudomonas aeruginosa*, but not by *Staphylococcus aureus*. Importantly, A101 increased the aminoglycosides antibiotics' capability of killing *P. aeruginosa* biofilm. Cell primary attachment to surfaces and intercellular aggregates assays suggested that A101 inhibited cell aggregates of both *P. aeruginosa* and *S. aureus*, while the cell-surface interactions inhibition only occurred in *S. aureus*, and the pre-formed cell aggregates dispersion induced by A101 only occurred in *P. aeruginosa*. Taken together, these data identify the antibiofilm activity of A101, which may make it potential in the design of new therapeutic strategies for bacterial biofilm-associated infections and limiting biofilm formation on medical indwelling devices. The found of A101 antibiofilm activity may also promote a new recognition about the functions of bacterial exopolysaccharides.

## Introduction

Bacterial biofilms are dense aggregates of cell–cell or surface-attached microorganisms encased in a hydrated extracellular polymeric substances matrix of self synthesized [1], [2]. Bacterial pathogens living in biofilm induce persistent chronic infections due to the resistance to antibiotics and host immune system as a result of the diffusion barrier, metabolism slowly, genetic mutant, biofilm specific phenotypes, persistent cells and so on [3], [4]. Compared with the planktonic ones, bacteria within the biofilm state are upward of 1000-times more resistant to conventional antibiotic treatment and host immune responses [5], [6]. Recent estimates suggest that bacterial biofilms are accounting for over 80% of microbial infections in the body [3], [7]. Therefore, searching for compounds or strategies to decrease bacterial biofilm formation or the antibiotics resistance of the pathogens in biofilms, is very essential and useful for the treatment of biofilm-associated disease.

The traditional approach to prevent biofilm formation *in vivo* is local administration of biocides [8]. However, the bacterial biofilms often persist in the presence of large doses of traditional antimicrobial agents [9]. Recently various approaches were proposed and expected to be effective in directly preventing or eliminating bacterial biofilms. One of common effective strategies is directly targeting at the component of biofilm. For example, the enzymes to degrade extracellular matrix or exopolysaccharide (EPS) were shown not only to inhibit biofilm formation, but also to remove pre-existing biofilms effectively, although the effects depend on the specificity of EPS matrix composition of biofilms [10]–[13].

EPS is a common component of biofilm and its production is an important feature of the mature biofilm [5]. In many bacteria, increased biofilm formation often correlates with increased EPS production. During the process of biofilm formation, by using EPS glycocalyx polymers, bacterial cells initiate the adhesion to the surface and the development of microcolonies [14]–[17]. In addition, EPSs form the matrix that embeds the bacteria, where additional free bacteria can be entrapped [18], [19]. However, few bacterial EPSs were recently found to negatively regulate biofilm formation. Capsular polysaccharide (CPS) transportation protein gene mutant in *Vibrio vulnificus* [20] or a putative glycosyltransferase gene deletion in *Porphyromonas gingivalis* [21] was found to decrease the production of EPSs and enhance biofilm formation of themselves. Jaione Valle el. at reported that *E. coli* group II capsular polysaccharide exerted broad-spectrum biofilm inhibition activity; however, it had no effect on the established biofilms [22]. In addition, *P. aeruginosa* extracellular products, mainly polysaccharides, were found to disrupt the established *S. epidermidis* and *S. aureus* biofilms [23], but biofilms of other pathogens are not involved.

In this study, we showed that an exopolysaccharide A101 purified from culture supernatant of the marine bacterium *Vibrio* sp. QY101 not only inhibited biofilm formation by a wide range of Gram-negative and Gram-positive bacteria, but also disrupted the established biofilms of some strains. Furthermore, A101-mediated biofilm disruption significantly decreased the minimum biofilm eradication concentration (MBEC) of antibiotics. And the mechanism underlying the antibiofilm effect of A101 was preliminarily investigated. This is the first reported bacterial EPS that exhibits both biofilm formation inhibition activity and pre-existing biofilm disruption activity.

## Results

### *Vibrio* sp. QY101 culture supernatant inhibits biofilm formation of *P. aeruginosa* FRD1

**Figure 1:**
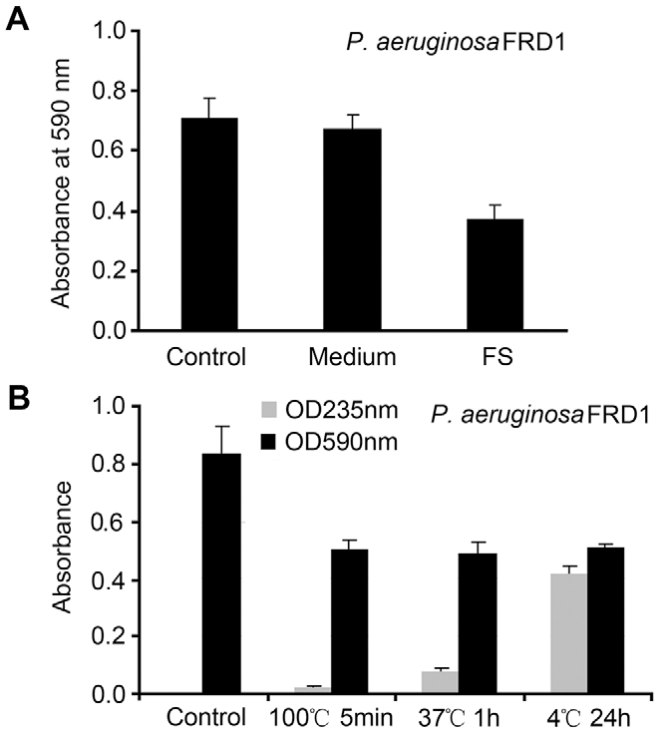
Effect of *Vibrio* sp. QY101 supernatant on biofilm formation of *P. aeruginosa* FRD1. *Vibrio* sp. QY101 was grown in AM at 25°C for 4 days. Its supernatant was taken to examine the effect on bacterial biofilm formation. *P. aeruginosa* FRD1 biofilm formed in micro-tubes at 37°C for 24 h. Attached biofilm biomass was determined by measuring crystal violet retained at OD590 nm. Results are the average of six replicates ± SD at least three independent experiments. (A) 1/10 (v/v) supernatant was added in the cultures. (*P*<0.01). Control: sterile water; Medium: alginate medium; FS: fermentation supernatant. (B) The supernatant was treated to make the alginate lyase inactive before being added in the cultures. Alginate lyase activity was measured by an increase in absorbance at 235 nm of the reaction products.

The alginate lyase-producing marine bacterium *Vibrio* sp. QY101 was isolated from a decaying thallus of *Laminaria* [24]. Here its culture supernatant was discovered to inhibit *P. aeruginosa* FRD1 biofilm formation, which was not caused by the original medium (Figure 1A). It has been reported that the acetylated alginate was very important for *P. aeruginosa* FRD1 biofilm formation [25]. Therefore, we speculated that the biofilm inhibitory activity of the culture supernatant was due to the alginate lyase activity. However, it was found that the purified alginate lyase from *Vibiro* sp. QY101 had no biofilm inhibitory activity (data not shown), and the culture supernatant still inhibited *P. aeruginosa* FRD1 biofilm formation after loss of enzyme activity (Figure 1B), demonstrating the active factor was not alginate lyase.

**Figure 2:**
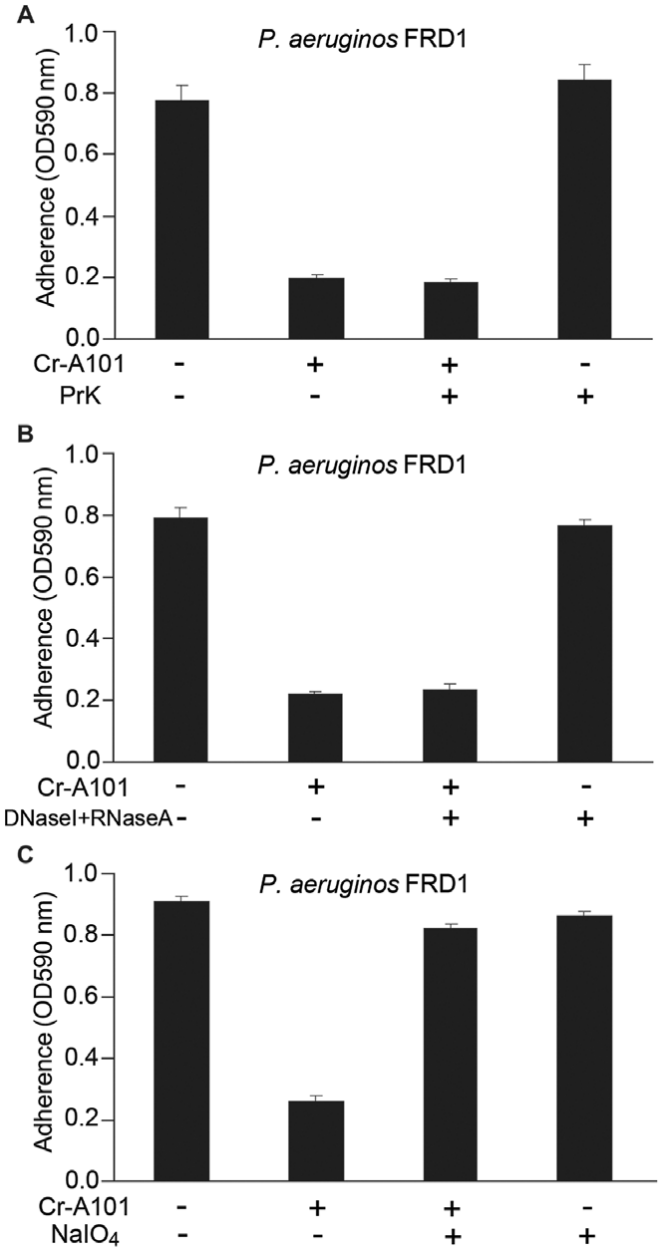
Effect of Proteinase K, Nuclease and NaIO_4_ on the activity of A101. Crude-A101 (150 mg/ml) was respectively treated with proteinase K (1 mg/ml) (A), DNaseI (100 µg/ml) + RNaseA (25 µg/ml) (B) or NaIO_4_ (20 mM) (C) at 37°C for 12 h, then taken to measure the antibiofilm activity. The strain used in this experiment was *P. aeruginosa* FRD1. Error bars represent SD of three independent experiments. “Cr-A101”: Crude-A101.

To elucidate the active component, ethanol precipitation, dialysis and freeze-dried were applied to extract the active factor from the supernatant of *Vibrio* sp. QY101. Then proteinase K, nuclease and NaIO_4_, were used to digest the crude extract (crude-A101), respectively. As shown in Figure 2A and 2B, pretreatment crude-A101 with proteinase K and nuclease had no effect on the antibiofilm activity, while pretreatment with NaIO_4_ resulted in a more than 60% decrease (Figure 2C). It is well known that NaIO_4_ is able to hydrolyze polysaccharides by oxidizing the carbons bearing vicinal hydroxyl groups and cleaving the C-C bonds [15], [26]. These data suggested that the biologically active component was likely a polysaccharide.

### Isolation and purification of the active polysaccharide

The crude polysaccharides were extracted from the supernatant of QY101 and then were further purified by DEAE Sepharose Fast Flow column. The fraction eluted by 0.5 mol/L NaCl contained abundant active sugars (Figure S1A). The active fraction was pooled, dialyzed, lyophilized and was further fractionated on a Sephacryl S-400 HR column eluting with 0.2 mol/L ammonium bicarbonate (Figure S1B). Finally, one active polysaccharide was obtained and named as A101.

### IR spectra and chemical compositions of A101

Fourier transform infrared spectra (FT-IR) of A101 revealed typical characteristics of polysaccharides, which displayed a stretching vibration at 3417 cm^−1^ for the hydroxyl group and a weak C–H stretching band at 2925 cm^−1^. The characteristic absorption at 1634 cm^−1^ was also observed, suggesting the presence of C-O bonds, whose symmetrical stretching vibration was at 1416 cm^−1^. The signals at 1040–1082 cm^−1^ were attributed to the stretch vibration of C–O and change angle vibration of O–H.

High performance liquid chromatography (HPLC) traces of the polysaccharide hydrolyzates showed monosaccharide components of A101. As shown in Table S1, the predominant monosaccharides of A101 were glucuronic acid (21.47%), galacturonic acid (23.05%), rhamnose (23.90%) and glucosamine (12.15%). Besides the four abundant monosaccharides, mannose, glucose, galactose and fucose were also present in A101 at low level. Additional, high performance gel permeation chromatography (HPGPC) was applied to elucidate the molecular weight (Mw) of the polysaccharide. One independent and symmetrical peak was identified and the average Mw value of A101 is about 546 KDa.

### The inhibitory activity of A101 on biofilm formation does not result from reducing bacterial growth

**Figure 3:**
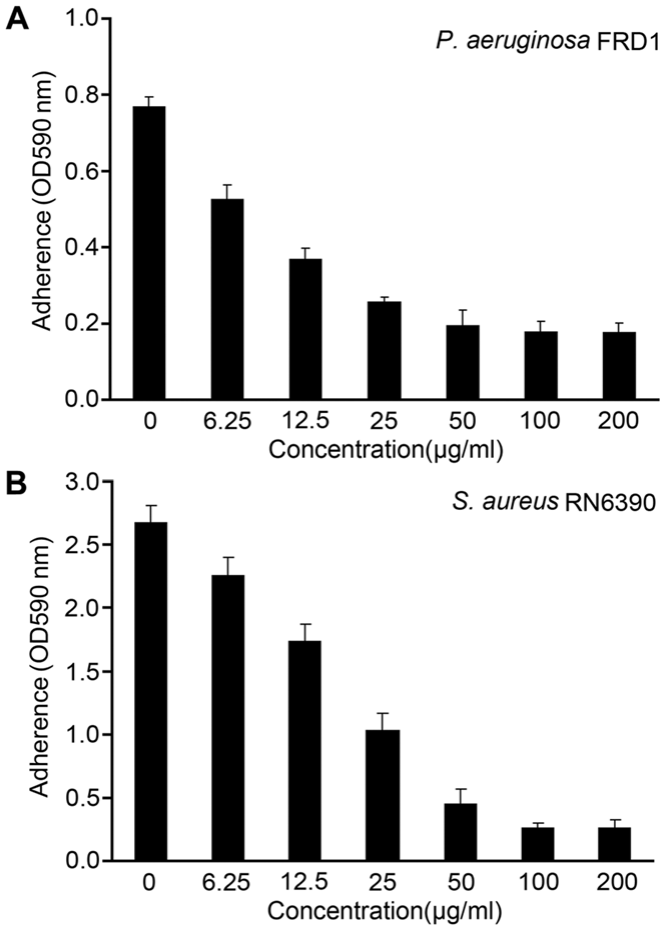
A101 inhibits bacterial biofilm formation in a concentration dependent manner. Biofilms of *P. aeruginosa* FRD1 (A) and *S. aureus* RN6390 (B) were grown in 96-well microplates containing medium at 37°C for 24 h. Medium was mixed with A101 of different concentrations in a does-dependent manner. Results are the average of six replicates ± SD.

In the microtiter plates biofilm assays, A101 displayed a dose-dependent inhibitory effect on biofilm formation of *P. aeruginosa* FRD1 and *S. aureus* RN6390 (Figure 3). In the presence of 100 µg/ml A101, about 75% of biofilm formation of *P. aeruginosa* FRD1 was inhibited (Figure 3A), and *S. aureus* RN6390 revealed >90% inhibition (Figure 3B).

**Figure 4:**
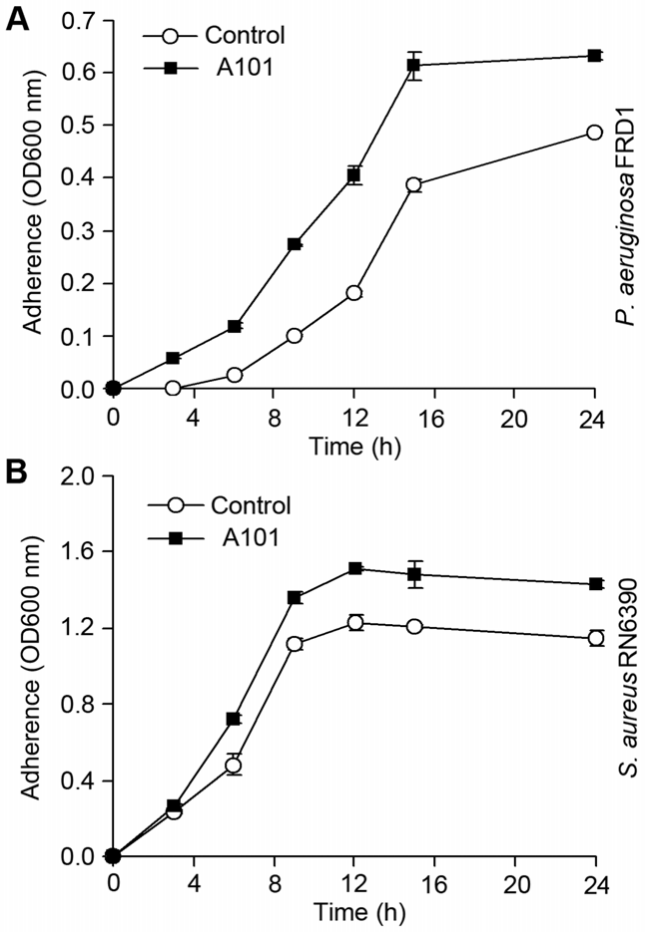
Growth curve of planktonic bacteria in the presence or absence of A101. *P. aeruginosa* FRD1 (A) and *S. aureus* RN6390 (B) were incubated in fresh medium with or without (Control) 100 µg/ml A101 at 37°C for 24 h with shaking. At regular intervals, 200 µl cultures were taken to measure the absorbance at OD600 nm. Graph show the mean of three experiments; error bars show SD.

To investigate whether the inhibitory effect of A101 on bacterial biofilm formation might due to the direct growth inhibition, growth curve analysis was performed. The results revealed that both *P. aeruginosa* FRD1 (Figure 4A) and *S. aureus* RN6390 (Figure 4B) grew a bit faster in the presence of 100 µg/ml A101, indicating that A101 was not bactericidal.

### The inhibitory activity of A101 on biofilm formation occurs among multiple strains

**Figure 5:**
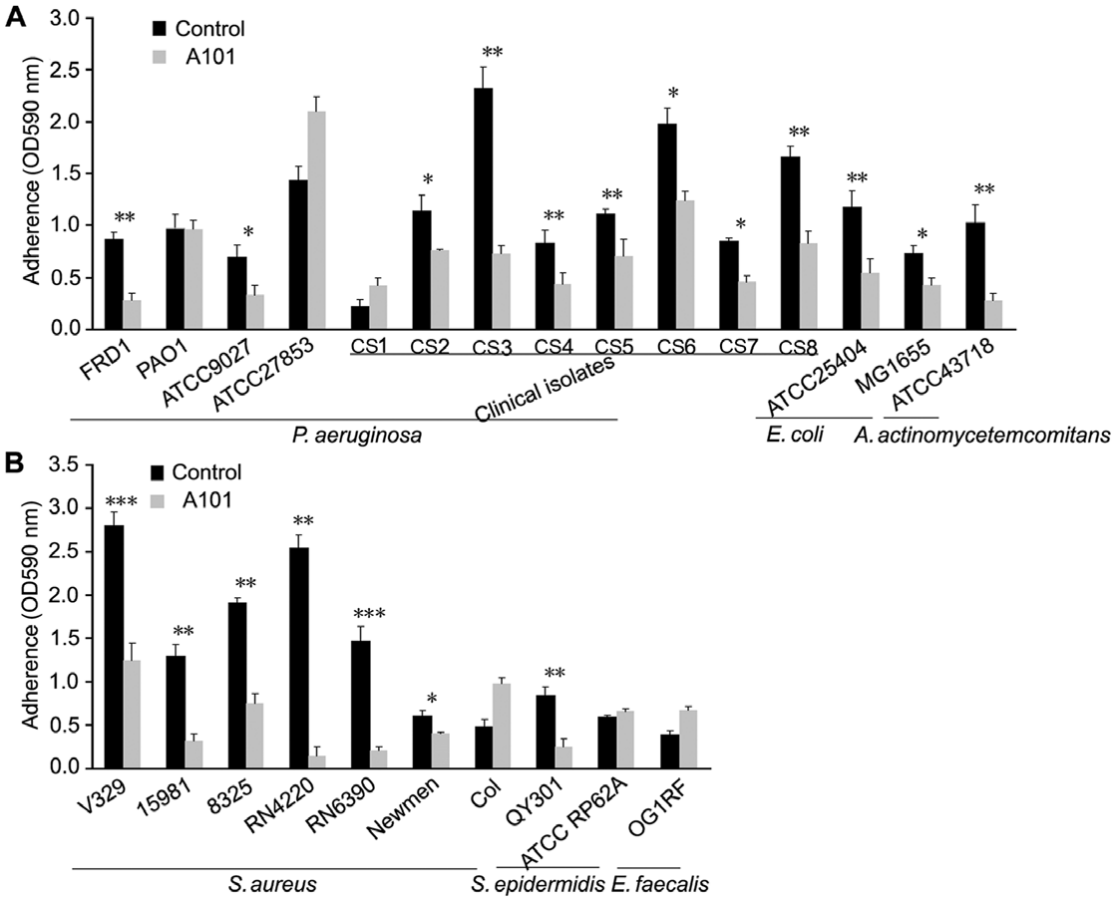
Effect of A101 on Gram-negative and Gram-positive bacteria biofilm formation. Biofilms of Gram-negative (A) and Gram-positive (B) bacteria formed in 96-well containing fresh medium with or without (Control) 100 µg/ml A101 at 37°C for 24 h. Levels of crystal violet retained were measured spectrophotometrically (OD590). Results are the average of six replicates ± SD at least three independent experiments. “*”, *P*<0.05; “**”, *P*<0.01; “***”, *P*<0.001.

To determine the spectrum of A101 antibiofilm activity, the effects of A101 on biofilm formation of various strains (*P. aeruginosa, E. coli, Actinobacillus actinomycetemcomitans, S. aureus, S. epidermidis, Enterococcus faecalis*) were tested using the microtiter plate assay. As indicated in Figure 5, biofilms formation by 12 out of 15 Gram-negative strains (80%) and 7 out of 10 Gram-positive strains (70%) can be inhibited by A101 under static condition, although 4 tested strains' biofilm formation was stimulated (*P. aeruginosa* ATCC27853 and CS1, *S. aureus* Col and *E. faecalis* OG1RF). Furthermore, A101 exhibited prominent prevention effects (≥50%) on 12 out of 25 tested strains (48%). These data demonstrated the antibiofilm activity of A101 occurred across multiple strains of Gram-negative and Gram-positive bacteria.

**Figure 6:**
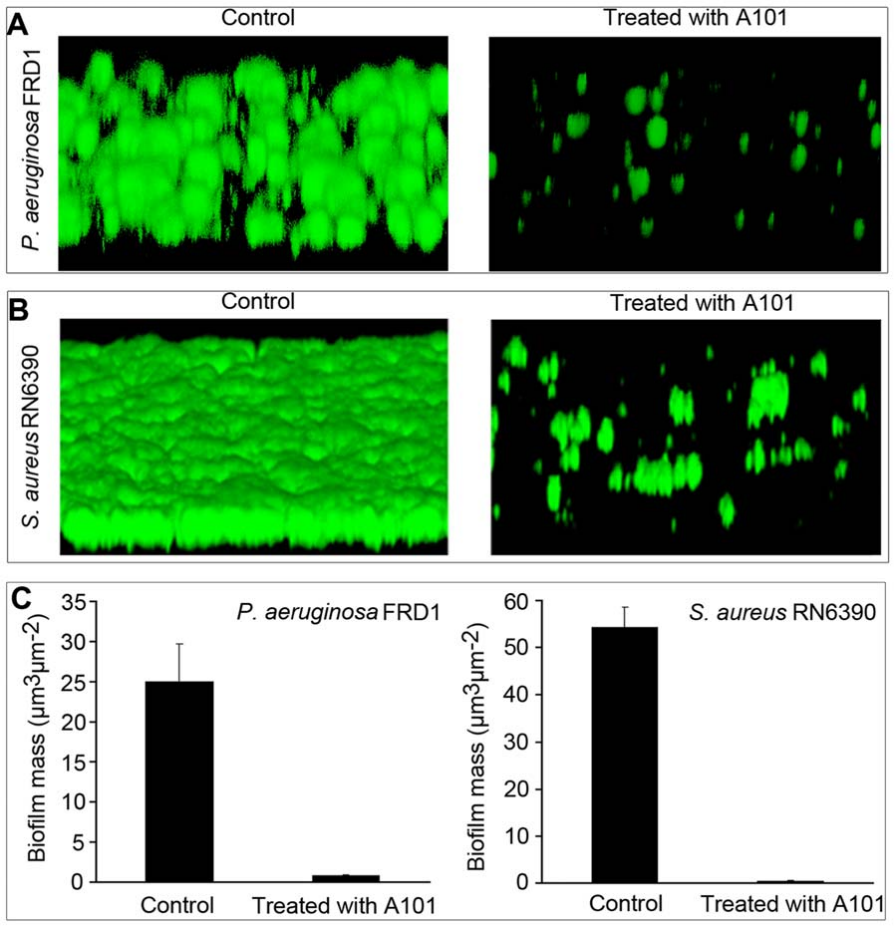
Prevention biofilm formation with A101 in flow cell. Flow cells irrigated by medium with and without (Control) 100 µg/ml A101 were inoculated with *P. aeruginosa* FRD1 at 25°C for 2 days (A) or *S. aureus* RN6390 at 37°C for 24 h (B). Images and structures of biofilms in flow cells were detected by CLSM. CLSM images are representative of three separate experiments. Biomass of biofilm in flow cells were analyzed using COMSTAT software (C). (*P*<0.001).

Although the polystyrene microtiter plate assay is a simple means of testing bacterial biofilm formation, it measures biofilm formation in static cultures but not in a naturally hydrodynamic environment. To better demonstrate the effect of A101 on bacterial biofilm formation, we used a flow cell model that allows the continuous flow of fresh nutrients into a chamber. The GFP-tagged *P. aeruginosa* FRD1 and *S. aureus* RN6390 were grown in flow cell in the absence or continued presence of A101, and biofilm was subsequently analyzed using confocal laser scanning microscopy (CLSM). The results showed that A101 treatment dramatically reduced biofilm development of both strains (Figure 6A and 6B). Analysis using COMSTAT software showed that in the presence of 100 µg/ml A101, the total surface-bound biomass of *P. aeruginosa* FRD1 dropped to less than 5% of that grown in the absence of A101 (Figure 6C Left), while the same treatment resulted in a more than 99% decrease to the surface-bound biomass of S. *aureus* RN6390 (Figure 6C Right). These findings demonstrated that A101 also had biofilm inhibitory activity in flow cell with dynamic medium that is similar to the models *in vivo*.

### A101 disrupts the established bacterial biofilm

**Figure 7:**
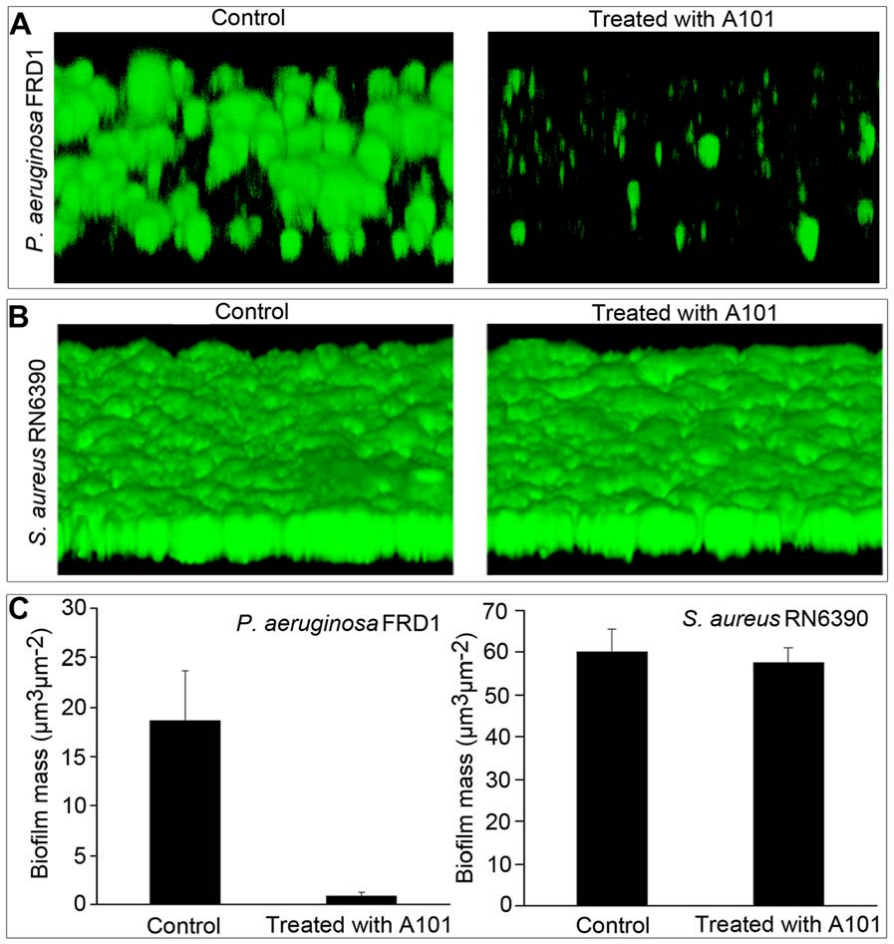
Disruption of the established biofilm with A101 in flow cell. Biofilm of *P. aeruginosa* FRD1 in flow cells was preformed at 25°C for 2 days (A) and *S. aureus* RN6390 at 37°C for 24 h (B). Then mediums were switched to fresh ones with or without (Control) 100 µg/ml A101 for an additional 12 hours. Images and structures of biofilms in flow cell were detected by CLSM. CLSM images are representative of three separate experiments. Biomass of biofilm in flow cells were analyzed using COMSTAT software (C). (*P*<0.001).

While A101 exhibited an inhibitory activity against bacterial biofilm formation, it was of interest to explore whether the established biofilms were also sensitive to A101. This assay was performed by culturing the strains in flow-cell model and irrigated with medium in absence of A101 to allow the establishment of mature bacterial biofilms. Subsequently, the bacterial biofilms were shifted to medium supplemented with A101, and the fate of the bacterial biofilms was followed by CLSM. The results showed that the pre-existing biofilms of *P. aeruginosa* FRD1 were disrupted severely when treated with 100 µg/ml A101 (Figure 7A Right). Analysis using COMSTAT software suggested that about 85% of mature biofilms were disappeared (Figure 7C Left). Strangely, A101 couldn't eliminate the mature biofilms of *S. aureus* RN6390 at all (Figure 7B, Figure 7C Right).

### A101 increases the antibiotics' capability of killing *P. aeruginosa* FRD1 biofilm

A marker signifying bacterial biofilm production in pathogens is the development of resistance to antibiotics [3], [27]. To investigate whether A101-mediated biofilm disruption increased the antibiotic sensitivity of bacterial biofilm, the effect of A101 on MBEC of antibiotic was examined. *P. aeruginosa* FRD1 biofilm, similar to previous reports, even at the highest concentration tested (2048 µg/ml) were not killed by amikacin. However, the viability of *P. aeruginosa* FRD1 biofilm displayed a different antibiotic response in the presence of A101 (100 µg/ml) that addition of amikacin (128 µg/ml) resulted in complete killing of the biofilm (Table 1). Moreover, no synergy was detected between A101 and the used antibiotic on planktonic *P. aeruginosa* FRD1, because minimal inhibitory concentration (MIC) and minimal bactericidal concentration (MBC) tests of amikacin resulted in the same value in the presence and absence of A101 (data not shown). These results suggested that A101-mediated biofilm dispersion increased the antibiotic's capability of killing bacterial biofilms by indirectly targeting to bacterial cells.

**Table 1:** Antibiotic sensitivity of *P. aeruginosa* FRD1 as a planktonic population (MIC and MBC) and as a biofilm population (MBEC).

Antibiotics	MIC (µg/ml)	MBC (µg/ml)	MBEC (µg/ml)	
			Control^a^	A101 (100 µg/ml)
Amikacin	16	32	>2048	128
Tobramycin	4	8	512	16
Gentamicin	16	32	2048	64

a The equal volume of sterile water was as control.

Results shown are representative of six replicates at least three independent experiments.

### A101 inhibits cell-surface adherence and intercellular adhesion

**Figure 8:**
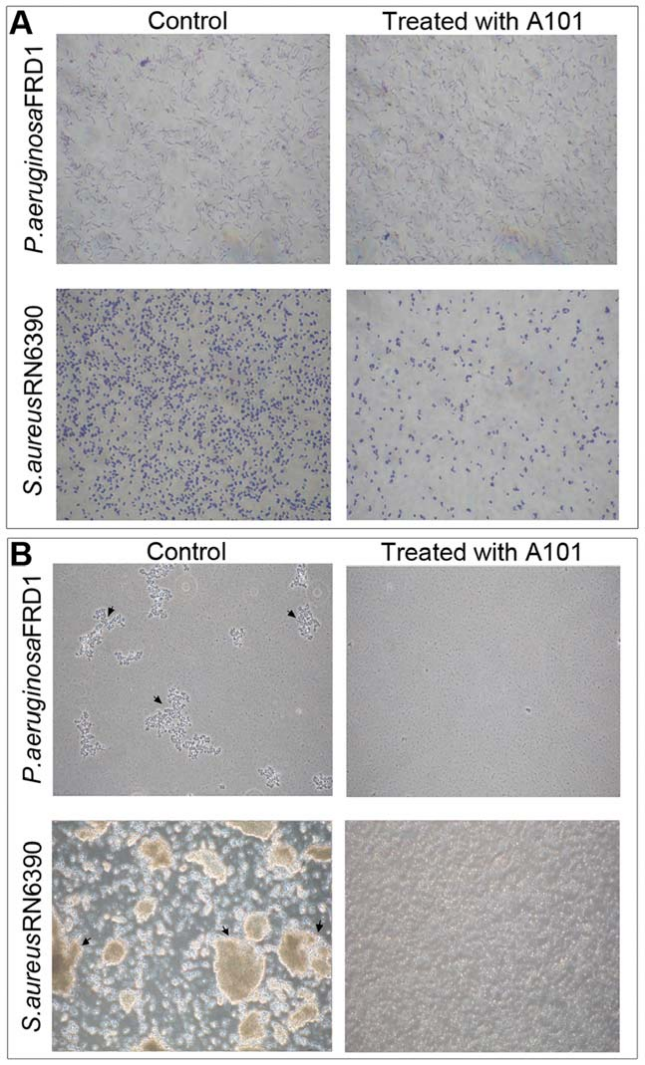
Inhibition of A101 on the early adherence to surfaces and cell aggregates of strains. (A) Detection the numbers of *P. earuginosa* FRD1 (Up) or *S. aureus* RN6390 (Down) attached on the surfaces with (Right) or without (Left) (Control) 100 µg/ml A101. The numbers of adherent cells to the surfaces were detected by phase-contrast microscopy ×600. (B) Observation the formation of aggregates for *P. earuginosa* FRD1 (Up) or *S. aureus* RN6390 (Down) in cultures with (Right) or without (Left) (Control) 100 µg/ml A101. Strains were cultivated in medium (M9 (FRD1) or 20% TSB+0.25% glucose (RN6390)) with or without A101 at 37°C for 24 h with shaking, and then observed. The numbers of cells aggregates in cultures were detected by phase-contrast microscopy ×200.

It is well established that cell-surface interactions are critical for bacterial biofilm formation. To investigate the effects of A101 on the cell surface interactions, the primary adherence to abiotic surfaces assay was performed. The initial attachment of *P. aeruginosa* FRD1 to surface displayed no difference in the presence (100 µg/ml) and absence of A101 (Figure 8A Up), whereas significant decreases in the adhesion of *S. aureus* RN6390 were observed when 100 µg/ml A101 was present (Figure 8A Down). This demonstrated that A101 inhibiting celll-surface interactions occurred on *S. aureus* RN6390, but not on *P. aeruginosa* FRD1.

Generally, cell-cell interactions also significantly contribute to bacterial biofilm formation. *P. aeruginosa* FRD1 and *S. aureus* RN6390 often grow in an aggregated state in cultures, forming readily observable clumps (the planktonic biofilm) [28] (Figure 8B Left). However, when A101 was present in medium, the aggregates of *P. aeruginosa* FRD1 and *S. aureus* RN6390 were powerfully decreased (Figure 8B Right), suggesting that A101 was able to interfere with the cell-cell interactions and inhibit multicellular aggregates formation of the both strains. And to further observe the effects of A101 on the preformed aggregates, A101 was added to the overnight cultures of strains and incubated for 3 hours. The results showed that the percentage of *P. aeruginosa* FRD1 but not *S. aureus* RN6390 in the cultures that were present in aggregates were decreased by A101 (data not shown).

## Discussion

To our knowledge, the biodiversity of the marine environment and the associated chemical diversity constitute a practically unlimited resource of new bioactive substances [29], and marine diversity is also a potent source of identifying novel antibiofilm compound(s) [30]. In this study, the marine bacterium *Vibrio* sp. QY101 was found to secret an antibiofilm exopolysaccharide A101.

Disrupting the multicellular structure of bacterial biofilm was proposed one of the most potential strategies for increasing the sensitivity of pathogens in biofilm to antibiotics and host immune systems [31], such as enzymes dissolving the matrix polymers of the biofilm [13], [32], chemical reactions blocking biofilm matrix synthesis, and analogues of microbial signaling molecules interfering with cell-to-cell communication, required for normal biofilm formation [33], [34]. Here, A101 was demonstrated to powerfully disrupt the multicellular aggregates and established biofilm of *P. aeruginosa* FRD1, and significantly increased the aminoglycosides antibiotics' capability of killing *P. aeruginosa* FRD1 biofilm. As *P. aeruginosa* could cause acute and chronic lung infections and result in significant morbidity and mortality in cystic fibrosis [35], the effectiveness of A101 in inhibiting biofilm growth in a majority of *P. aeruginosa* strains suggest that A101 might be a powerful therapeutic tool in the treatment of *P. aeruginosa* infections. That biofilm formation of few tested strains was not inhibited by A101 may be related with the complex and probably multifactorial mechanisms involving in biofilms formation [36], [37]. And similar observation has been reported that *P. aeruginosa* (mainly extracellular polysaccharides) exhibited different effects on biofilm formation of different *S. epidermidis* strains [23], [38]. In addition, the exopolysaccharide A101 displayed inhibitory activity towards biofilm formation of many Gram-negative and Gram-positive bacteria, including clinic isolates. A101 is potentially superior to other antibiofilm substances in a multispecies biofilm context.

Based on our findings, the antibiofilm activity of A101 is independent of decreasing bacterial viability. Instead, A101 promoted the bacterial growth a little. However, further investigation indicated that strains did not have the ability to utilize the polysaccharide A101 as carbon source, because strains were not able to grow in medium with A101 as the only carbon source (data not shown). And the mechanisms about the A101 promotion bacterial growth are needed to further investigate.

Bacterial extracellular polysaccharides synthesized and secreted by a wide range of bacteria from various environmental habitats are complex and diverse. Some have been proved to involve the pathogenicity [39], [40], while some were found to serve a structural role benefitting the bacterium by providing protection from antimicrobial agents and host defenses [16], [41]–[43], helping nutrient acquisition [44], or promoting adherence to surfaces [45]–[47] and biofilm formation [17], [48]. A101 and other few bacterial EPSs were found to negatively regulate biofilm formation [20]–[23]. As it is known, polysaccharides functions are based on their divergent compositions, biochemical characteristics and structures [20], [49]. Previous studies showed that EPSs in *V. cholerae* are one important component of its biofilm [50], [51], which primarily contain neutral sugars glucose and galactose [52]. While *E. coli II* CPS [53] and *V. vulnificus* CPS [54] with antibiofilm activity were indicative of highly anionic nature. And A101 also displayed high content of uronic acid sugars and electronegative, although its monosaccharides compositions exhibited complexity. These data suggested that the electronegative uronic acid sugars in A101 may play the crucial role for antibiofilm activities.

Cell-surfaces and cell-cell interactions play an important role in bacterial biofilm formation [55]. Many active compounds secreted by bacteria were reported to mediate the interaction (adhesion and deadhesion) between microorganisms and interfaces [49]. And receptor polysaccharides recognized by a complementary protein adhesin were reported to mediate the establishment of productive cell-to-surface and cell-to-cell contacts [56]–[58]. Recent reports demonstrated that polysaccharide-probes have the potential to interfere with adherence and biofilm formation [59]. The *E. coli* group II CPS was also reported to inhibit biofilm formation not only by weakening cell-surface contacts but also by reducing cell–cell interactions [22]. In this study, A101 was indicated to inhibit or disrupt the interactions of cell-surfaces and cell-cell. It is thus likely that A101-mediated inhibition or disruption cell-surface and cell-cell interactions induced the antibiofilm effects. Moreover, we found that the complete hydrolysis of A101 lost the antibiofilm activity. It is proposed that A101 may contain the motif recognized by adhesins, which caused competitively blocking the recognition of adhesins and receptor polysaccharides on strains surfaces and interfering with the adherence of cell-surface and cell-cell. However, investigation the possible binding of A101 with adhesins would be needed to further understand the mechanism underlying the antibiofilm effects.

A101 was suggested here to inhibit the cells aggregates formation of both *S. aureus* RN6390 and *P. aeruginosa* FRD1, but only disrupt the mature biofilm of *P. aeruginosa* FRD1. And further investigation demonstrated that A101 was only able to disrupt the formed cells aggregates of *P. aeruginosa* FRD1, but not that of *S. aureus* RN6390 (data not shown). It has been reported that binding of phage 1P to *R. leguminosarum* bv. *Trifolii* involved a reversible binding to EPS and an irreversible binding to the specific lipopolysaccharides receptors [60], [61]. We proposed that the antibiofilm activity of A101 may be involved irreversible binding or adsorption in *S. aureus* RN6390, and reversible in *P. aeruginosa* FRD1. Moreover, previous findings that the three-dimensional structure and matrix composition of biofilm were physical barriers to the penetration of antimicrobial agents [3], led us to hypothesis that the different activity against established biofilms of the two strains could also be related with the different architecture and matrix composition of both biofilms: A101 maybe easily penetrate in *P. aeruginosa* biofilms, but not penetrate in *S. aureus* biofilms.

To conclude, our findings suggest that A101 is of significance in controlling biofilm-associated infections, even in a multispecies biofilm context, and the antibiofilm activity appears to represent a new function of bacterial EPSs. However, the antibiofilm activity of A101 warrants further investigations of the structure–activity relationships of A101 and the mechanism underlying the antibiofilm effects.

## Materials and Methods

### Strains and growth conditions

All strains and plasmids used in this study were listed in Table 2. *P. aeruginosa* was cultured in M9 medium; *S. aureus*, *S. epidermidis, E. faecalis*, and *A. actinomycetemcomitans* were grown in Tryptone Soya Broth (TSB) (Sigma) supplemented with 0.25% glucose; and *E. coli* was cultured in Luria-Bertani (LB) medium (Sigma). The marine bacterium *Vibrio* sp. QY101 was cultured in alginate medium (AM) (w/v, 3% NaCl, 0.5% sodium alginate (Sigma), 0.3% KH_2_PO_4_, 0.7% K_2_HPO_4_, 0.2% (NH_4_)_2_SO_4_, 0.01% MgSO_4_, 0.01% FeSO_4_) at 25°C to prepare A101. All the reagents used here are analytical grade.

**Table 2:** Strains and plasmids list.

Strains or plasmids	Relevant characteristics	Source or reference
*Pseudomonas aeruginosa*		
FRD1	A cystic fibrosis isolate mucA22	[76]
PAO1	Prototroph, nonmucoid ATCC 15692	[77]
9027	A clinical isolate recovered from an ear infection	ATCC^a^
27853	A clinical isolate from blood	ATCC^a^
CS1-8	Clinical strains	AHQU^b^
*Escherichia coli*		
25404	A wide K-12 strain of *E. coli*	[78]
MG1655	A wild-type laboratory strain of *E. coli* K-12	[78]
*Actinobacillus actinomycetemcomitans* 43718	The smooth laboratory strain Y4	ATCC^a^
*Staphylococcus aureus*		
V329	A bovine mastitis subclinical isolate	[71]
15981	A clinical isolate	[71]
8325	A strain with rsbU^−^, tcaR^−^	[71]
RN4220	A restriction-deficient mutant of 8325-4	[71]
RN6390	A laboratory strain derived from 8325-4	[79]
Newmen	A human clinical isolate	[71]
Col	A methicillin-resistant isolate	[71]
*Staphylococcus epidermidis*		
QY301	A clinical strain	AHQU^b^
RP62A	A laboratory strain	ATCC^a^
*Enterococcus feacalis* OG1RF	A laboratory strain	[80]
*Vibrio* sp. QY101	A isolate from a decaying thallus of *Laminaria*	This work
Plasmids		
pMRP9-1	Car resistance	[62]
pSB2019	Chl resistance	[63]

a American Type Culture Collection.

b Affiliated Hospital of Qingdao University.

Plasmids pMRP9-1-GFP (carbenicillin 200 µg/ml) [62] and pSB2019-GFP (chloromycetin 15 µg/ml) [63] were transformed into appropriate strains by electroporation for biofilm formation experiments in flow cell. The software COMSTAT 7.0 was a kind gift from Dr. Arne Heydorn and used to analyze the biofilm parameters [64], [65].

### Isolation and purification of A101

Polysaccharides preparation was carried out as previously described [66], [67]. *Vibrio* sp. QY101 cells were grown in AM with shaking at 25°C for 4 days. The culture supernatant was concentrated under reduced pressure at 40°C, and the EPS was precipitated by adding threefold of the volume of 95% (v/v) ethanol. After keeping at 4°C overnight, the precipitate was dried in a Speed Vac concentrator and dissolved in distilled water followed by dialysis through a 14,000-molecular-weight cutoff membrane. The retained fraction was recovered, concentrated under reduced pressure. After removing proteins by Sevage method, it was lyophilized to obtain the crude polysaccharide. The solution of crude polysaccharide was applied to a DEAE Sepharose Fast Flow column (2.6×50 cm) (Pharmacia) and was eluted by a step gradient of NaCl concentration (0–2 mol/L) at a flow rate of 60 ml/h. Total sugar content of the elution was determined by the phenol-sulfuric acid method. The fractions eluted by 0.5 mol/L NaCl were pooled, desalted and loaded on a Sephacryl S-400 HR column (2.6×100 cm) (Pharmacia) equilibrated with 0.2 mol/L ammonium bicarbonate. The elution was done with the same solution at a flow rate of 12 ml/h, and the active polysaccharide fractions were concentrated under reduced pressure named as A101.

### Analysis of the infrared spectra and chemical composition of A101

For IR spectroscopy, as previously described [68], A101 was mixed with KBr powder, ground and then pressed into a 1 mm pellets for FT-IR measurement in the frequency range of 4000–500 cm^−1^. FT-IR spectra of A101 was measured on a Nicolet Nexus 470 spectrometer.

Total sugar content was determined by the phenol–sulfuric acid method using glucose as the standard [69]. Protein content was measured by the method of BCA. The molecular weight were assessed by HPGPC [68]. The monosaccharide components were determined by HPLC[68]. The standard saccharides used here were glucose, galactose, mannose, rhamnose, xylose, fucose, arabinose, glucuronic acid, galacturonic acid and glucosamine.

### Biofilm assay

Static biofilm formation assay was carried out in 96-well microtiter plates (Costar, Cambridge, MA, USA) or mircrotiter tubes as previously described [70], [71]. Briefly, overnight cultures were diluted to about 1×10^6^ cfu/ml with fresh sterile medium, and each well of microtiter plates was filled with 100 µl aliquots of the diluted cultures. The plates were incubated at 37°C for 24 h without shaking for biofilm growth, and non-adherent bacteria were washed by 0.9% (w/v) NaCl for three times. Biofilm was strained by 0.1% (w/v) crystal violet solution for 10 min, washed and then dissolved with 33% (v/v) acetic acid for 10 min. Biofilm mass was determined by measuring the absorbance at 590 nm using a microtiter plate reader (Tecan, Milan, Italy).

Dynamic biofilm formation analysis was carried out using a “once-through” flow cell (4.5 mm×2 mm×35 mm) model [70], [72]. Each cell was injected with 0.8 ml of the diluted culture of the tested strain, and incubated for 40 min without flow. Non-adherent bacteria were then flushed from the flow cell by adjusting the pump to a flow rate (4 ml/h for *P. aeruginosa* [70], 21 ml/h for *S. aureus* [72]). CLSM was performed as previously described [73]. The images were constructed with the LSM 5 image browser.

### Antibiotics susceptibility assay of bacteria and biofilm

MIC, MBC and MBEC were performed as previously described [74]. MICs of antibiotics were determined using the broth microdilution method. Briefly, the test antibiotic was diluted with MHB medium (Sigma) from 1024 µg/ml to 2 µg/ml by 2-fold serial dilutions. The final concentration of bacterial cultures was about 10^5^ cfu/ml. After incubation at 37°C for 24 h, the absorbance at 600 nm was measured using a microplate reader to assess the cell growth. To assay the MBC, 100 µl cultures was taken from each well in the above MIC experiment, centrifuged and washed three times with PBS and plated on a LB agar plate separately, and bacterial cells were enumerated after incubation at 37°C for 24 h. MBEC™ (MBEC Bio-Products, Edmonton, Canada) assays were performed here to detect tolerance to antibiotics of biofilm bacteria. The device used here is a platform carrying 96 polystyrene pegs that fit in a microtiter plate. For biofilm formation, the device was placed in a tray filled with MHB and cells (∼10^7^ cfu/ml) and was incubated on a tilting shaker, which provides a shearing force, at 37°C for 22 h. After biofilms formed on the pegs, non-adherent bacteria on the pegs were washed from the pegs in a 96-well microtiter plate containing sterile 0.9% (w/v) NaCl. Pegs with the bacterial biofilm were placed into a new microtiter plate with fresh medium and incubated at 37°C for 24 h with antibiotic, which was diluted from 1024 µg/ml to 2 µg/ml. The remaining biofilm was transferred from the pegs into a second 96-well microtiter plate containing fresh broth medium by being sonicated for 5 min in a water bath sonicator. This plate was incubated at 37°C for 24 h. Growth of bacteria in a particular well indicates regrowth of planktonic bacteria from surviving biofilm.

### Early bacterial adhesion assay

Early adherence to a polystyrene surface was analyzed as previously described [75] with the following modifications. Overnight cultures of tested strains were grown in medium supplemented with or without A101 at 37°C for 12 h, then adjusted with fresh sterile medium with or without A101 to an optical density at 600 nm of 0.1. Each suspension was added to 24-well microtiter plates (Costar, Cambridge, MA, USA) and incubated at 37°C for 1 h. The 24-well microtiter plates were rinsed gently at least six times with sterile phosphate buffer solution (pH 7.5) and strained by 0.1% crystal violet for 20 min. Adherent bacterial cells were observed by phase contrast microscopy and counted. The results represent the means of four different microscopic fields.

## Supporting Information

Figure S1 **Purification of the polysaccharides obtained from marine bacterium** ***Vibrio*** **sp. QY101.** (A) A mixture of polysaccharides obtained from the marine bacterium *Vibrio* sp. QY101 was applied to a DEAE Sepharose Fast Flow column and eluted as described in Method. The fractions containing the active polysaccharides were pooled and named as pre-A101. (B) pre-A101 obtained on DEAE Sepharose Fast Flow was applied to a Sephacryl S-400 HR column and eluted as described in Method. The fractions containing the active polysaccharides were pooled and named as A101.
(TIF)

Table S1 **The monosaccharides compositions and the average Mw of A101.**
(DOC)
