# Supplementary figures and images for: Antibiofilm Activity of an Exopolysaccharide from Marine Bacterium Vibrio sp. QY101
Source: PLoS One. 2011 Apr 7;6(4):e18514. doi: 10.1371/journal.pone.0018514 (PMC3072402; doi:10.1371/journal.pone.0018514)

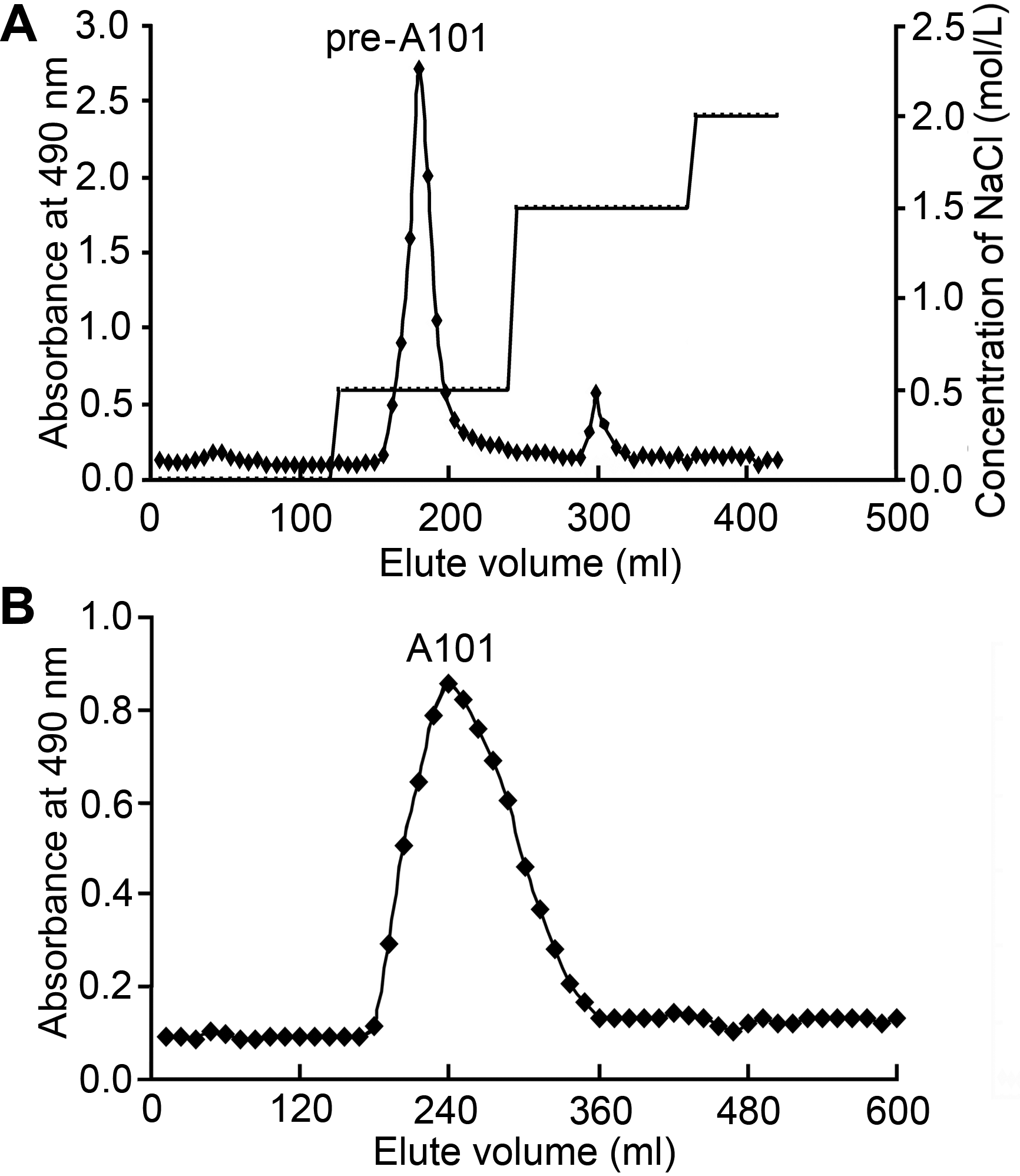

Supplement: Figure S1 — Purification of the polysaccharides obtained from marine bacterium Vibrio sp. QY101. (A) A mixture of polysaccharides obtained from the marine bacterium Vibrio sp. QY101 was applied to a DEAE Sepharose Fast Flow column and eluted as described in Method. The fractions containing the active polysaccharides were pooled and named as pre-A101. (B) pre-A101 obtained on DEAE Sepharose Fast Flow was applied to a Sephacryl S-400 HR column and eluted as described in Method. The fractions containing the active polysaccharides were pooled and named as A101. (TIF) [file pone.0018514.s001.tif]
