# Supplementary material for: Antibiofilm Activity of an Exopolysaccharide from Marine Bacterium Vibrio sp. QY101
Source: PLoS One. 2011 Apr 7;6(4):e18514. doi: 10.1371/journal.pone.0018514 (PMC3072402; doi:10.1371/journal.pone.0018514)
Supplement: Table S1 — The monosaccharides compositions and the average Mw of A101. (DOC) [file pone.0018514.s002.doc]

**Table S1. The monosaccharides compositions and the average Mw of A101.**

| **Glycosyl residue** | **Mol%** |
| --- | --- |
| Glucose | 6.57 |
| Galactose | 6.89 |
| Glucuronic acid | 21.47 |
| Galacturonic acid | 23.05 |
| Rhamnose | 23.90 |
| Fucose | 3.61 |
| Glucosamine | 12.15 |
| Mannose | 2.36 |
| Mw | 546 KDa |
